# Gastrointestinal Adverse Effects of Anti-Obesity Medications in Non-Diabetic Adults: A Systematic Review

**DOI:** 10.3390/medicina61111987

**Published:** 2025-11-05

**Authors:** Ehab Takrori, Supriya Peshin, Sakshi Singal

**Affiliations:** 1 College of Medicine, Alfaisal University, Riyadh 11533, Saudi Arabia; etakrori@gmail.com; 2 Department of Internal Medicine, Norton Community Hospital, Norton, VA 24273, USA; 3 Department of Hematology and Oncology, East Tennessee State University, Johnson City, TN 37614, USA; singal@mail.etsu.edu

**Keywords:** anti-obesity drugs, adverse effects, non-diabetic adults, toxicity, therapeutic use

## Abstract

*Background*: With rising obesity rates, pharmacological interventions are increasingly used in non-diabetic adults. While being effective in managing weight, these agents frequently cause gastrointestinal (GI) side effects, affecting adherence and long-term outcomes. *Objective*: To systematically evaluate the frequency, severity, and types of GI adverse effects (AEs) associated with anti-obesity medications in obese adults without diabetes. *Methods*: Following PRISMA 2020 guidelines, PubMed, Google Scholar, BMJ, and Web of Science were searched (last search July 2025). Eligible studies included randomized controlled trials, non-randomized trials, cohort studies, cross-sectional, and case–control studies. Only reports of GI AEs in non-diabetic adults were included. Risk of bias was assessed using Cochrane RoB 2 and Newcastle–Ottawa scales. *Results*: Out of 733 articles screened, 12 studies met predefined inclusion criteria, including one large cohort of 18,386 participants, along with randomized and observational trials of smaller size. The most frequently reported GI symptoms were nausea, vomiting, diarrhea, and constipation, predominantly with GLP-1 receptor agonists such as semaglutide and tirzepatide, especially during dose escalation. Orlistat commonly linked to steatorrhea and flatulence, while phentermine was associated with reduced GI motility. Newer agents, including retatrutide and orforglipron, also demonstrated notable GI side effect profiles. Natural products and investigational agents reported fewer adverse events but lacked long-term data and standardized reporting. *Limitations*: Evidence was limited by heterogeneity in study design and inconsistent reporting of GI outcomes. *Conclusion*: GI side effects are common across anti-obesity medications, particularly GLP-1 receptor agonists. Although generally mild to moderate, these symptoms can impact adherence and lead to treatment discontinuation. Tailored titration schedules, proactive patient counseling, and standardized adverse event reporting may improve tolerability. Further research is warranted to evaluate long-term GI outcomes and compare safety across emerging pharmacologic agents.

## 1. Introduction

With obesity rates growing, anti-obesity drugs are frequently utilized for weight control in adults without diabetes, even though their long-term side effects are yet to be investigated. According to the World Health Organization (WHO), over 1 billion people worldwide were obese in the year 2022, including 650 million adults [1]. Importantly, most of these individuals are non-diabetic, underscoring the urgent need to understand the safety of weight loss medications in this large population subset. Several anti-obesity medications have been approved by the U.S. Food and Drug Administration (FDA), including orlistat, phentermine-topiramate, naltrexone-bupropion, liraglutide, semaglutide, and tirzepatide, while newer agents such as orforglipron and retatrutide continue to emerge through late-phase trials [2,3,4,5,6,7]. These medications work through diverse mechanisms such as slowing of gastric emptying (GLP1 agonists), central sympathomimetic appetite suppression (phentermine), hypothalamic reward modulation (naltrexone/bupropion), and lipase inhibition (orlistat) [6,8,9,10,11].

Weight loss achieved by these agents is clinically impactful; however, gastrointestinal (GI) adverse effects (AEs) remain the leading cause of early discontinuation. Multiple trials reported the most commonly observed AEs included nausea, diarrhea, constipation, and abdominal discomfort [3,6,9,12,13]. Incretin-based therapies (semaglutide, liraglutide, exenatide) showed clear dose-dependent increase in nausea and diarrhea across both trials and real-world datasets [6,8,9,10,11]. Less effective drugs for weight loss, such as sympathomimetics and lipase inhibitors, were associated with more subtle AEs including oily stools and fecal urgency [3,14].

Recent investigations have also expanded into populations beyond those with diabetes, highlighting a critical gap in the evaluation of safety in non-diabetic adults. Importantly, medications such as phentermine, tirzepatide, and newer agents like S-309309 and vutiglabridin are increasingly used in non-diabetic individuals, yet comprehensive assessments of their GI safety profiles remain limited [2,3,15,16]. Additionally, some natural or complementary products, such as berberine, silibnin, and Lipigo, have been studied for weight loss, with reported side effects ranging from bloating and flatulence to more clinically significant GI disturbances [14,17].

Despite growing clinical experience, the long-term GI tolerability of these agents, especially in individuals without diabetes, has not been clearly synthesized. While GI symptoms are often listed in trials, they are variably reported and rarely stratified by severity of duration, making it difficult for clinicians to anticipate or manage such events across drug classes.

This systematic review aims to consolidate and evaluate the GI adverse effects associated with anti-obesity medications in non-diabetic adults. By examining primary studies, including randomized controlled trials and high-quality pharmacologic investigations, this review will provide an evidence-based summary of GI symptom patterns, severity, and frequency across drug classes. The goal is to inform clinical decision-making, identifying gaps in reporting, and guide future safety-focused research on weight-loss therapies.

## 2. Methodology

This systematic review was conducted in accordance with the Preferred Reporting Items for Systematic Reviews and Meta-Analyses (PRISMA) 2020 guidelines and prospectively registered in PROSPERO (CRD420251113277) [18]. The registration included the predefined objectives, eligibility criteria, data sources, and analysis framework, which served as the guiding methodological plan for this review. A separate detailed protocol document was not prepared, and no amendments were made to the registration.

### 2.1. Databases and Search Strategies

The literature search was conducted across four reputable databases: PubMed, Google Scholar, the British Medical Journal (BMJ) archive, and Web of Science. Search strategies were tailored to each platform and incorporated Medical Subject Headings (MeSH), advanced filtering options, and free-text keyword searches. The inclusion of BMJ was not intended as a database search but as a targeted manual review of the BMJ collection (specifically BMJ Open, BMJ Medicine, and BMJ Nutrition, Prevention and Health) to identify relevant clinical trials and reviews that may not yet have been indexed in major databases.

A condensed overview of search terms and filters with the full Boolean search strings are included in Table 1.

### 2.2. Eligibility Criteria

To ensure data relevance, a research question was developed using the PICO-TT framework, targeting non-diabetic obese individuals using anti-obesity agents and monitored for gastrointestinal adverse effects. The review included randomized controlled trials, non-randomized trials, cohort studies, cross-sectional studies, case–control studies, and interventional clinical trials, while excluding books, gray literature, and non-peer-reviewed sources. Only English-language articles published between 2020 and 2025 were considered. The inclusion and exclusion criteria are summarized in Table 2.

**Figure 1:**
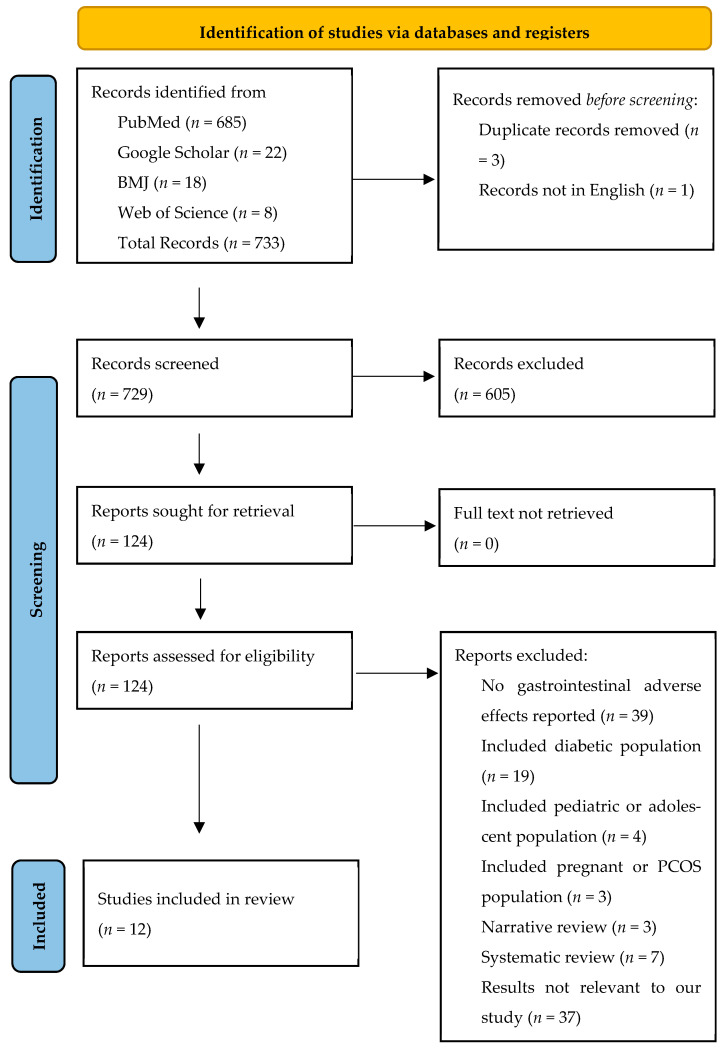
PRISMA Flow Diagram. BMJ: British Medical Journal; n: Number; PCOS: Polycystic Ovarian Syndrome; PRISMA: Preferred Reporting Items for Systematic Reviews and Meta-Analysis.

Based on these criteria, studies were screened according to titles, abstracts, and full texts as outlined in the PRISMA 2020 flow diagram (Figure 1). A total of 733 records were identified across databases, of which 12 studies met all inclusion criteria and were included in the final synthesis. The most common reasons for exclusion were the absence of gastrointestinal adverse effect data, inclusion of diabetic or adolescent populations, and non-interventional study designs such as narrative reviews or case reports. These criteria ensured that only peer-reviewed interventional studies in non-diabetic adults (≥18 years) evaluating gastrointestinal adverse effects of anti-obesity medications between 2020 and 2025 were retained.

### 2.3. Data Collection and Screening Process

All articles retrieved were imported into EndNote, and duplicates were removed. Titles and abstracts were screened independently by two reviewers to determine initial eligibility. Full texts were then assessed for inclusion. Any disagreements were resolved through consensus.

### 2.4. Data Extraction and Quality Assessment

Verifying the relevance of studies to the review topic involved a structured assessment of each article’s methodological quality and risk of bias (RoB) using established appraisal tools. These included the Cochrane Risk of Bias Tool for randomized trials and the Newcastle–Ottawa Scale for other studies as shown in Table 3 and Table 4. Each tool consists of a series of quality criteria, typically ranging from four to six items, used to evaluate the rigor and transparency of study design and reporting. The total scores were converted into percentage values, with articles scoring above 70% considered eligible for inclusion. Studies scoring below this threshold were excluded.

### 2.5. Data Preparation

No data conversion or imputations were required. Data were extracted as reported in the original studies and presented in descriptive tables and narrative synthesis.

### 2.6. Tabulation and Visual Display

Results of included studies were tabulated to summarize study design, population, interventions, and gastrointestinal outcomes. Narrative descriptions and summary tables were used to highlight similarities and differences across studies with results discussed in Section 4.

### 2.7. Synthesis Methods

A quantitative meta-analysis was not feasible due to heterogeneity in study design, outcome definitions, and reporting methods. Therefore, no statistical models for effect estimation, heterogeneity assessment, subgroup analysis, or sensitivity analysis were conducted. Instead, findings were synthesized narratively to identify common gastrointestinal adverse events and trends across drug classes.

### 2.8. Reporting Bias Assessment

Reporting bias was not formally assessed as unpublished data and trial registries were outside the scope of this review and the synthesis was descriptive in nature.

### 2.9. Certainty Assessment

Certainty of evidence was not formally graded using frameworks such as GRADE due to the heterogeneity and limited number of included studies. Instead, overall confidence was inferred from the risk of bias assessments and study quality ratings presented in Table 3 and Table 4.

## 3. Results

The PRISMA 2020 flow diagram (Figure 1) provides a detailed summary of the screening and selection process. A total of 733 records were identified, of which three duplicates and one record not in English were removed. After screening 733 titles and abstracts, 605 were excluded. From this screening, 124 full text articles were assessed and 112 were excluded for the following reasons: no GI adverse effects reported (*n* = 39), studies contained diabetic adults (*n* = 19), studies included pediatric and adolescent individuals (*n* = 4), studies included pregnant individuals or those with polycystic ovarian syndrome (PCOS) (*n* = 3), narrative reviews (*n* = 3), systematic reviews (*n* = 7), outcomes not relevant to our study (*n* = 37), and studies using animal models (*n* = 1). Twelve studies met all eligibility and quality criteria and were included in the final synthesis.

Figure 1 provides a detailed overview of the screening and selection process, including the number of excluded articles at each stage. Table 3 and Table 4 present the quality appraisal results of the included articles according to the applied assessment tools. The PRISMA 2020-based diagram is presented below in Figure 1.

### 3.1. Summary of Findings by Drug Class

A synthesized overview of GI adverse effects reported by drug class is presented in Supplementary Table S1. This table includes commonly reported symptoms, approximate frequency where available, and the pooled sample sizes from the relevant studies.

The most frequent and severe GI symptoms were associated with GLP-1 receptor agonists, particularly during dose titration phases. These symptoms were often transient but contributed to early treatment discontinuation in several studies. Orlistat was less likely associated with nausea; however, it frequently contributed to steatorrhea. Natural products generally showed better tolerability profiles, though the sample sizes and methodological rigor in those trails were comparatively lower.

### 3.2. Narrative Summary of Evidence Certainty (GRADE Approach)

Following the quality appraisal (Table 3 and Table 4), a narrative GRADE-style summary of evidence certainty for GI AEs across all included studies is presented in Table 5. This table qualitatively synthesizes the overall confidence in the evidence for each pharmacological class, based on the consistency, reporting quality, and methodological rigor of the included trials. The certainty of evidence ranged from low to moderate, reflecting strong consistency among incretin-based therapies but variable reporting standards and smaller sample sizes in other drug classes.

Evidence certainty was appraised narratively according to GRADE domains (risk of bias, inconsistency, indirectness, imprecision, and publication bias). Risk of bias was evaluated using the Cochrane RoB 2 and the Newcastle–Ottawa assessments (Table 3 and Table 4). Publication bias was not formally tested because of qualitative synthesis. Overall, the certainty of evidence for gastrointestinal (GI) adverse effects (AEs) ranged from low to moderate, with highest consistency among incretin-based therapies.

### 3.3. Summary of Gastrointestinal Adverse Effects Across Studies

Across the twelve included trials, GI AEs represented the most frequent treatment-emergent events, though their frequency and severity varied among pharmacologic classes (Supplementary Table S1).

#### 3.3.1. GLP-1 Receptor Agonists (Semaglutide, Liraglutide, Exenatide, Orforglipron)

In the STEP 1 trial, semaglutide recipients experienced GI disorders in 74.2% versus 47.9% with placebo. Nausea occurred in 44.2% vs. 17.4%, diarrhea 31.5% vs. 15.9%, vomiting 24.8% vs. 6.6%, and constipation 23.4% vs. 9.5%. Serious GI adverse events included cholelithiasis (1.8%) and pancreatitis (0.2%) versus 0.6% and 0% in placebo, respectively. Discontinuation due to GI events occurred in 4.5% on semaglutide vs. 0.8% on placebo [13].

In STEP 4, diarrhea occurred in 14.4% of semaglutide-treated participants vs. 7.1% placebo, nausea 14.0% vs. 4.9%, and constipation 11.6% vs. 6.3%; discontinuation rates for GI events were 2.4% vs. 2.2%, respectively [6].

In Lundgren et al., liraglutide monotherapy produced nausea (65%), vomiting (22%), diarrhea (27%), and constipation (18%), while rates were lower with exercise + liraglutide or placebo [10]. Rodgers et al. reported exenatide-related nausea in 70% vs. 25% of controls and decreased appetite in 41% vs. 24%; no serious GI events occurred [9]. Klausen et al. observed GI side effects in 44.1% of exenatide vs. 23.6% of placebo participants [20]. In Wharton et al. (orforglipron 12–45 mg) nausea occurred in 37–58% vs. 10% placebo, vomiting 14–32% vs. 6%, diarrhea 3–36%, and constipation 13–32%; discontinuation due to GI effects ranged from 10 to 17% across dose groups [7].

Across all GLP-1 receptor agonist studies, most symptoms were mild to moderate and dose-dependent, peaking during escalation and resolving with continuation.

#### 3.3.2. Dual and Triple Incretin Agonists (Tirzepatide and Retatrutide)

The Rodriguez et al. conducted a comparative analysis between semaglutide and tirzepatide (*n* = 18,386) and found similar event rates per 1000 person-years: bowel obstruction 6.26 vs. 5.54 (HR 1.12; 95% CI 0.63–1.97), cholecystitis 6.5 vs. 5.06 (HR 1.25; 95% CI 0.70–2.21), cholelithiasis 11.89 vs. 12.66 (HR 0.94; 95% CI 0.63–1.39), and gastroenteritis 19.75 vs. 20.07 (HR 1.00; 95% CI 0.73–1.37) [8]. In SURMOUNT-4, diarrhea occurred in 10.7% vs. 4.8%, nausea 8.1% vs. 2.7%, vomiting 5.7% vs. 1.2%, and discontinuation due to GI events was 1.8% vs. 0.9% for tirzepatide vs. placebo [12]. In SURMOUNT-CN, diarrhea occurred in 40.0% and 40.8% at 10 mg and 15 mg vs. 8.7% placebo; nausea 30.0% and 32.4% vs. 5.8% placebo; vomiting 11.4% and 19.7% vs. 4.3% placebo. Events clustered during dose escalation [11]. The review by Jeon et al. confirmed that nausea, diarrhea, vomiting, constipation, and dyspepsia were the most frequent tirzepatide-related AEs [5].

For retatrutide, nausea ranged from 14 to 60%, vomiting 3–26%, diarrhea 9–20% vs. placebo 11%, 1%, and 11%, respectively. Discontinuation due to AEs occurred in 6–16% of treated vs. none on placebo. ALT elevations >3 times the upper limit of normal occurred in 1%, resolving spontaneously; one case of acute pancreatitis was reported [4].

#### 3.3.3. Sympathomimetic and Combination Therapies

In Márquez-Cruz et al., phentermine 15 mg produced abdominal pain in 0.5%, constipation 3.5%, dry mouth 6.3%, nausea 0.5%, vomiting 0.3%; at 30 mg the rates were 2.4%, 4.1%, 13.9%, 1.1%, and 0.8%, respectively [3]. AEs were mild (82.1%) or moderate (17.9%); dropout rate was 8.4% at 3 months and 66.6% at 6 months [3]. Combination therapy with topiramate produced similar GI profiles dominated by constipation, dry mouth, and dysgeusia [2,5]. For naltrexone/bupropion, pooled COR-I/II/BMOD data indicated nausea, constipation, vomiting, and dry mouth as the most frequent GI AEs [5].

#### 3.3.4. Lipase Inhibitors

Orlistat was consistently associated with oily stools, fecal urgency, and flatulence with discharge, occurring in 15–30% of users [2,5,20]; fat soluble vitamin malabsorption and rare cases of gallstones, kidney stones, liver injury, and pancreatitis were noted as well. In Mártinez et al., silibinin produced fewer GI events than orlistat and was well tolerated in short-term randomized trials [21].

#### 3.3.5. Natural and Investigational Agents

In Valero-Pérez et al., bloating occurred in 24.3% vs. 2.9% placebo at week 2, with no other significant GI differences and no withdrawals due to GI events [17]. In Bandala et al., Gymnema sylvestre caused loose stools (52%), dysgeusia (40%), diarrhea (28%), reflux (28%), nausea (20%). Berberine caused hemorrhoidal bleeding (28%), nausea (20%), constipation (16%); however, all symptoms were mild and self-limited [14]. The review by Sayed et al. highlighted scarce quantitative GI data for other botanicals such as green tea catechins, capsaicin, Garcinia cambogia, and mulberry leaf polyphenols [22]. Cabrera-Rode et al. observed mild, transient nausea, flatulence, and abdominal discomfort with Obex, without serious AEs [19]. Won et al. reported vutiglabridin-related nausea in 25% of participants, resolving spontaneously [15]. Ishibashi et al. reported mild diarrhea and reflux (<10%) and no serious events for S-309309 [16].

#### 3.3.6. Overall Summary

Across all pharmacologic classes, GI AEs were most frequent during dose-escalation periods, generally mild to moderate and resolved spontaneously. Discontinuation due to GI events ranged roughly 1–17%, highest with high-dose incretin-based therapies. Gallbladder-related events were uncommon (<2%), but slightly more prevalent with incretin drugs. Natural and investigational agents produced the fewest reported GI events but are supported by small, short-term studies, limiting generalizability.

## 4. Discussion

This systematic review highlights that GI AEs are the most frequent treatment-emergent effects across pharmacologic weight loss therapies in non-diabetic adults. Nearly all included agents, particularly those acting on the incretin pathway, were associated with varying degrees of GI intolerance, typically emerging during dose escalation and declining over time. Although generally mild to moderate, such adverse effects remain clinically important, as they can influence adherence and long-term treatment success.

A detailed summary of GI AEs frequencies by drug class is presented in Section 3.3. The following discussion interprets these findings in the context of underlying mechanisms, class-specific tolerability, and clinical implications.

### 4.1. Comparative Interpretation by Drug Class

#### 4.1.1. Incretin-Based Therapies

GLP-1 receptor agonists (semaglutide, liraglutide, exenatide, orforglipron) consistently produced the highest frequency of GI events among approved pharmacotherapies [6,7,9,10,13]. Their mechanism of delayed gastric emptying and central appetite suppression explains the predominant symptoms of nausea, diarrhea, vomiting, and constipation. These dose-dependent and transient effects represent expected pharmacologic consequences of GLP-1 receptor stimulation rather than direct toxicity.

Dual and triple incretin agonists (tirzepatide and retatrutide) showed similar profiles, suggesting a class effect rather than molecular-specific difference [4,8,11,12]. Despite high early-phase event rates, most studies reported low discontinuation (<7%) once maintenance dosing was reached. Gradual titration, slower escalation, and supportive management could substantially improve tolerability [11,12].

#### 4.1.2. Non-Incretin Pharmacotherapies

Sympathomimetic agents such as phentermine and combinations like phentermine/topiramate or naltrexone/bupropion, produced fewer GI events overall. The most common effects were mild constipation, dry mouth, or dysgeusia [2,3,5]. While their GI safety profile is favorable compared to incretin agents, these drugs may carry central nervous system or psychiatric side effects that limit long-term use.

#### 4.1.3. Lipase Inhibitors

Orlistat causes mechanism-specific adverse effects including steatorrhea, fecal urgency, and oily stools, due to fat malabsorption [2,5]. These events are non-systematic and manageable through dietary modification and vitamin supplementation. The plant-derived agent silibinin demonstrated fewer and milder GI events than orlistat in short-term trials [21], suggesting potential as a more tolerable lipase inhibitor, though evidence remains preliminary.

#### 4.1.4. Natural and Investigational Compounds

Agents such as Lipigo, Gymnema sylvestre, berberine, obex, and vutiglabridin demonstrated favorable short-term tolerability with only mild, transient GI events [14,15,17,19]. Likewise, S-309309 was associated with diarrhea and reflux in <10% of participants [16]. However, these studies were small, short in duration, and heterogenous in design. The evidence base for such natural and investigational agents remains limited compared to established pharmaceuticals.

### 4.2. Cross-Class Patterns and Mechanistic Insights

Across all drug classes, the timing of GI AEs strongly implicates the dose-escalation phase as the key driver of intolerance [6,7,13]. This temporal pattern supports the role of delayed gastric emptying and vagal stimulation as mechanistic pathways underlying nausea and early satiety in incretin-based therapies [8,11,12].

Gallbladder-related events and rare pancreatitis cases were more frequently observed with GLP-1 based drugs, consistent with their known effects on biliary motility and lipid metabolism rather than direct pancreatic toxicity [4,6,13]. Conversely, the local intestinal effects of orlistat and silibinin arise from luminal fat hydrolysis, while sympathomimetic drugs modulate central satiety pathways with minimal GI tract involvement [2,5].

These findings collectively indicate that the biological target of each drug class largely determines its GI tolerability profile, guiding clinicians in anticipatory counseling and dose adjustment strategies.

### 4.3. Clinical Implications

The comparative tolerability profile observed across trials translates directly into practical treatment decisions. GLP-1 receptor agonists remain the most effective for weight reduction but require proactive management of GI effects. Individualized titration schedules, reassurance about symptom transience, and short-term symptomatic therapy can maintain adherence and limit discontinuation to below 5–7% once maintenance dosing is achieved [6,11,12,13].

For patients unable to tolerate incretin-based therapy, phentermine/topiramate or naltrexone/bupropion combinations offer a lower GI burden but more modest efficacy [2,3,5]. Orlistat provides a non-systemic alternative, though adherence often depends on dietary compliance and counseling regarding expected oily stools and vitamin supplementation [2,5,21].

Natural and emerging compounds such as silibinin, Lipigo, Gymnema sylvestre, berberine, and vutiglabridin appear well tolerated in early studies, but their small sample sizes and short follow-up preclude strong conclusions [14,15,17,22]. They may serve as adjuncts or alternatives for patients unwilling or ineligible to use approved therapies but should not replace evidence-based medications until validated by larger randomized trials.

### 4.4. Cross-Class Consderation for Mitigation

The uniformity of GI-event patterns across incretin-based drugs indicates that titration speed is the key modifiable factor influencing early discontinuation. Regimens extending dose escalation beyond 4–8 weeks, temporary dose holds, and hydration or meal-timing adjustments can markedly improve tolerability [4,11,12]. Patient education and anticipatory counseling are also critical. Trials in which participants were pre-informed about likely GI symptoms, such as those by Rodgers et al. and Lundgren et al., reported higher adherence and lower dropout rates [9,10].

### 4.5. Mechanisms of Gastrointestinal Adverse Effects and Mitigation Strategies

The mechanisms responsible for GI intolerance to anti-obesity medications vary by pharmacologic class. For incretin-based agents, nausea and diarrhea are primarily mediated by delayed gastric emptying, activation of central satiety centers in the hypothalamus, and enhanced vagal signaling that collectively slow motility [4,6,8,11,13]. These effects peak during early dose escalation, when GLP-1 receptor activation exceeds adaptive accommodation; symptoms typically resolve as tolerance develops. For lipase inhibitors such as orlistat and silibinin, steatorrhea, flatulence, and fecal urgency result from unabsorbed triglycerides undergoing bacterial hydrolysis in the colon, leading to increased short-chain fatty acid and gas formation [2,21]. These effects are diet-dependent and predictable. Sympathomimetic and combination agents induce modest constipation or nausea through central norepinephrine and dopamine modulation that alters intestinal tone [3,5]. Natural and investigational products such as Gymnema sylvestre and berberine likely act through local modulation of gut microbiota and bile acid metabolism, producing mild, transient discomfort [14,22].

Mitigation strategies emphasized across trials include gradual titration, temporary dose holds, and supportive symptomatic therapy. Slower escalation over 8–12 weeks markedly reduces early-phase nausea in GLP-1 therapies [11,12]. Adequate hydration, small frequent meals, and avoidance of high-fat foods reduces nausea and bloating. Rodgers et al. showed that prophylactic antiemetic use significantly decreased exenatide-related nausea and vomiting, supporting the benefit of anticipatory management. For orlistat, dietary fat restriction and vitamin supplementation minimize steatorrhea and nutrient loss [2]. Patient education remains essential to maintain adherence and prevent premature discontinuation.

### 4.6. Comparison with the Broader Literature

Our findings in non-diabetic adults are consistent with larger synthesis including broader or mixed populations. Ismael et al. found that GLP-1 receptor agonists produced GI AEs in a dose-dependent manner, varying by agent and exposure [23]. Chiang et al. reported increased risks of cholelithiasis and gastroesophageal reflux disease (GERD), while other GI or biliary complications were not significantly elevated, indicating a selective safety signal [24]. Liu et al. confirmed consistent GI tolerability patterns across incretin drugs regardless of diabetic status, supporting a class effect [25]. McGown et al. observed that higher-efficacy incretin agents showed proportionally greater GI tolerance, mirroring our results [26]. Fredrick et al. described the expected profile of nausea, diarrhea, vomiting, and constipation with GLP-1 therapies and outlines management strategies aligning with our recommendations [27].

Beyond incretin-based therapies, broader reviews reported similar qualitative trends. McGown et al. noted that GI intolerance is most characteristic of lipase inhibitors such as orlistat, whereas combination agents, including phentermine/topiramate and naltrexone/bupropion, are generally associated with milder GI or oral symptoms such as constipation and dry mouth [26]. Fredrick et al. similarly emphasized that while non-incretin therapies produce fewer GI complaints, they achieve more modest weight loss. Both reviews highlight the limited safety evidence for natural or lipase-modulating products such as silibinin [26,27]. Together, these studies reinforce the gradient identified in our review, with GI AEs being most pronounced with incretin-based pharmacotherapies, moderate with lipase inhibitors, and mild with non-incretin and natural agents.

### 4.7. Limitations and Future Directions

This systematic review synthesized data qualitatively rather than through a formal meta-analysis. Although meta-analysis can provide pooled effect estimates, heterogeneity in study design, populations, AE reporting precluded reliable aggregation. Only a few studies explicitly addressed potential confounders. Rodriguez et al. adjusted for baseline factors such as age, body weight, and diabetic status while acknowledging unmeasured behavioral influences [8]. Ponce Martínez et al. standardized dietary variables to reduce confounding [21]. Most other trials, however, did not account for potential confounders such as preexisting gastrointestinal conditions, dietary patterns, or concomitant medication use, which may influence AE reporting.

A further limitation involves inconsistent symptom grading and patient-reported outcomes. Several trials incorporated validated health or quality-of-life instruments, including the 36-Item Short Form Health Survey (SF-36) or its version 2 and the Impact of Weight on Quality of Life-Lite Clinical Trials Version (IWQOL-Lite-CT) questionnaire [6,11,12,13], while Ishibashi et al. classified events using the Medical Dictionary for Regulatory Activities (MedDRA) terminology and Ponce Martínez et al. employed an ordinal appetite scale [16,21]. However, none used GI-specific severity frameworks such as Common Terminology Criteria for Adverse Events (CTCAE) nor evaluated satisfaction or adherence impact. Although a narrative GRADE-style summary (Table 5) was provided, formal GRADE scoring and publication bias testing were not feasible. Future systematic reviews with a larger number of homogenous randomized trials should include these analysis to improve evidence certainty. In addition, none of the included trials specifically assessed patient satisfaction, and no study used GI symptom-specific instruments to measure the impact of adverse effects on daily life. Future trials should incorporate validated GI-focused patient-reported outcome measures and treatment-satisfaction tools to enable meaningful cross-study comparisons and to better link tolerability with adherence and patient-centered outcomes.

Finally, evidence strength varied widely: while approved anti-obesity medications such as GLP-1 receptor agonists and combination therapies are supported by multiple large-scale randomized trials, natural and investigational products rely on limited short-term data. These results should thus be interpreted cautiously until confirmed by robust long-term studies.

## 5. Conclusions

Current evidence confirms a clear trade-off between weight loss potency and GI tolerability across pharmacologic anti-obesity agents in non-diabetic adults. Injectables and oral incretin-based therapies provide the greatest efficacy in weight reduction but are associated with the highest early phase rates of nausea, vomiting, and diarrhea, all of which can be attenuated, though not entirely eliminated, through gradual dose escalation and supportive management. Non-incretin oral combinations produce a lower GI burden but achieve more modest weight loss and may introduce stimulant or neuropsychiatric AEs. Mechanistic agents such as orlistat are primarily limited by predictable, diet-dependent effects like oily stools and reduced absorption of fat-soluble vitamins. Emerging natural and investigational compounds appear well tolerated in short-term studies but remain insufficiently characterized by large, long-term trials. Collectively, these findings highlight the importance of individualized drug selection, careful titration, and proactive management of GI symptoms to optimize adherence and therapeutic outcomes in weight loss pharmacotherapy. Future work should also prioritize the evaluation of patient satisfaction and GI specific quality-of-life outcomes to better inform tolerability-driven treatment choices.

## Figures and Tables

**Table 1:** Summary of Search Strategies Used Across Databases.

Databases	Keywords	Search Strategy	Filters	Search Result	Last Date Searched
PubMed (Advanced search)	Anti-obesity agents, Adverse effects (“Anti-Obesity Agents/adverse effects” [Majr] OR “Anti-Obesity Agents/therapeutic use” [Majr] OR “Anti-Obesity Agents/toxicity” [Majr])	Advanced filtered search and MeSH	5-year publication date	685	July 2025
Google Scholar (Advanced Search)	Anti-Obesity drugs	Advanced filtered search	5-year publication date, sort by relevance,	22	July 2025
BMJ	Anti-obesity agents, Adverse effects	Advances filtered search	5-year publication	18	July 2025
Web of Science	Anti-obesity agents, Adverse effects	Advanced filtered search	5-year publication, English, Full text	8	July 2025

MeSH: Medical Subject Heading; BMJ: British Medical Journal.

**Table 2:** PICO-TT framework.

Component	Inclusion	Exclusion
Population	Obese adults without diabetes	Young entity (<18 years), adults with diabetes
Intervention	Use of anti-obesity pharmacologic agents (e.g., Semaglutide, Orlistat, Setmelanotide, Phentermine-topiramate, Bupropion-naltrexone, Liraglutide, Tirzepatide, etc.)	Studies conducted in vitro or on animals
Comparison	Placebo or other anti-obesity agents (e.g., Glucomannan, garcinia combogia, herbal teas, herbal coffees, hoodia, chitosan, chromium picolinate, conjugated linolic acid, etc.)	
Outcome	Gastrointestinal adverse effects (e.g., delayed gastric emptying, nausea/vomiting, constipation/diarrhea, etc.)	Outcome other than gastrointestinal adverse effects
Timeframe	Studies published between 2020 and 2025	
Trial Type	Randomized controlled trials, non-randomized trials, cohort studies, cross-sectional studies, case–control studies, and interventional clinical trials	Systematic reviews, meta-analysis, case series, observational studies, editorials, books, and gray literature

**Table 3:** Cochrane Risk of Bias Assessment Tool.

	Randomization	Deviation from Intended Intervention	Missing Data	Measurement of Outcome	Reporting	Overall Percentage
Aronne et al., 2024 [12]	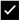	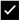	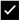	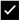	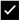	100%
Cabrera-Rode et al., 2023 [19]	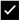	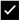	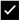	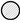	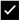	90%
Jasterboff et al., 2023 [4]	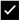	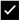	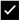	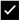	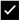	100%
Lundgren et al., 2021 [10]	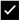	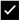	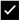	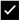	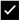	100%
Rodgers et al., 2021 [9]	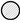	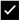	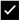	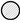	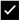	80%
Rubino et al., 2021 [6]	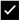	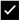	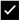	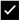	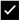	100%
Wharton et al., 2023 [7]	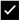	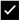	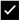	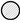	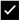	90%
Wilding et al., 2021 [13]	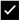	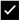	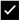	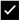	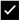	100%
Zhao et al., 2024 [11]	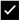	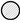	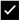	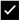	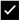	90%
Won et al., 2024 [15]	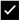	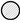	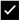	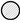	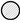	70%
Key:			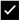	Low Risk of Bias		
			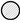	Some Concerns of Bias		

**Table 4:** Newcastle–Ottawa Quality Assessment Scale.

Author and Year of Publication	Type of Study	Selection (Maximum 4 Stars)	Comparability (Maximum 2 Stars)	Outcome (Maximum 3 Stars)
Márquez-Cruz, 2021 [3]	Cohort	****	**	***
Rodriguez, 2024 [8]	Cohort	****	**	***

Asterisks indicate points awarded per Newcastle-Ottawa Quality Assessment Scale domain.

**Table 5:** Narrative GRADE-style summary of evidence certainty for gastrointestinal adverse effects across anti-obesity drug classes.

Drug Class/Agents	Representative Studies	Summary of Findings on GI Adverse Effects	Certainty of Evidence (Narrative GRADE Assessment)	Key Limiting Factors
GLP-1 receptor agonists (Semaglutide, Liraglutide, Tirzepatide, Exenatide, Orforglipron)	Wilding et al. [13]; Rubino et al. [6]; Rodriguez et al. [8]; Zhao et al. [11]; Aronne et al. [12]; Lundgren et al. [10]; Rodgers et al. [9]; Wharton et al. [7]; Klausen et al. [20]	GI AEs were the most frequently reported, particularly nausea, diarrhea, vomiting, and constipation. Incidence was dose-dependent and highest during dose escalation. Most events were mild to moderate, transient, and led to discontinuation in 3–7% of participants. Gallbladder-related events (cholelithiasis, pancreatitis) occurred infrequently.	Moderate	Consistent findings across large RCTs but heterogeneity in AE definitions and absence of standardized GI severity grading; limited long-term tolerability data.
Sympathomimetic/Combination Therapies (Phentermine, Phentermine-Topiramate, Naltrexone-Bupropion)	Màrquez-Cruz et al. [3]; Jeon et al. [5]; Bays et al. [2]	Constipation, dry mouth, and nausea were the most common GI events. Events were generally mild, with low discontinuation rates. Some CNS-related side effects overlapped with appetite-suppressant mechanisms.	Low to Moderate	Small sample sizes; short follow up; limited GI-specific reporting; potential reporting bias in self-reported outcomes.
Lipase Inhibitors (Orlistat, Silibinin)	Martínez et al. [21]; Bays et al. [2]	Steatorrhea, fecal urgency, and flatulence with discharge were characteristic of orlistat use; silibinin showed fewer and milder GI events. Both agents showed predictable mechanism-related GI profiles.	Moderate	Limited sample sizes for silibinin; short-term studies; variation in AE definitions.
Triple-hormone receptor agonist (Retatrutide)	Jasterboff et al. [4]	GI AEs (nausea, vomiting, diarrhea) occurred in up to 82% of participants, primarily during dose escalation. Most events were transient; discontinuation due to GI effects ranged from 6–16%.	Moderate	Phase 2 data only; short follow-up; single study evidence.
Natural Agents/Botanicals (Lipigo, Gymnema sylvestre, Berberine)	Valero-Pérez et al. [17]; Bandala et al. [14]	Lipigo caused mild bloating in 24% of participants; Gymnema and Berberine caused mild diarrhea, nausea, or constipation, all self-limited. No severe or serious GI AEs reported.	Low	Small sample sizes; short study duration; lack of standardized AE reporting; potential publication bias.
Novel small-molecule agent (S-309309)	Ishibashi et al. [16]	GI symptoms reported in up to 25% of participants. Overall GI tolerability was good with no serious events.	Moderate	Early-phase data only; single small study; limited follow-up.
Multi-mechanism and emerging agents (Obex, natural product overviews, Vutiglabridin)	Cabrera-Rode et al. [19]; Sayed et al. [22]; Won et al. [15]	Across these heterogenous agents, GI events were generally mild and transient, including nausea, flatulence, or abdominal discomfort. Obex and Vutiglabridin demonstrated favorable tolerability profiles, while natural product reviews reported limited systemic AE data.	Low	Heterogenous interventions and study designs; small samples; lack of controlled comparison; incomplete AE reporting across studies.
Overall summary across all pharmacologic classes	-	Across all interventions, GI AEs were common but typically mild to moderate, transient, and related to mechanism of action. Rates of discontinuation due to GI events ranged from 3 to 16%, highest with incretin-based therapies.	Moderate	Heterogenous study designs, inconsistent adverse effect grading, and lack of gastrointestinal-specific patient reported outcomes limit comparability and confidence in pooled interpretation.

## Data Availability

All data supporting this review are contained within the article and its Supplementary Materials. This includes the PRISMA 2020 flow diagram, the PRISMA 2020 checklist, and extracted study tables. No additional datasets or analytic code were generated.

## Supplementary Materials

The following supporting information can be downloaded at: https://www.mdpi.com/article/10.3390/medicina61111987/s1. Table S1. Summary of Gastrointestinal Adverse Effects by Drug Class. This table provides an overview of the reported gastrointestinal adverse events, frequencies, and corresponding references for each pharmacological agent included in this review.
