# Supplementary material for: Gastrointestinal Adverse Effects of Anti-Obesity Medications in Non-Diabetic Adults: A Systematic Review
Source: Medicina (Kaunas). 2025 Nov 5;61(11):1987. doi: 10.3390/medicina61111987 (PMC12654588; doi:10.3390/medicina61111987)
Supplement: Supplementary file 1 [file medicina-61-01987-s001.zip › medicina-3909020-supplementary.pdf]

Supplementary Table S1. Summary of Gastrointestinal Adverse Effects by Drug Class.

Study	Drug Class	Agent(s)	Sample Size	Common GI AEs	Comparison	Comparison	Comparison	Comparison
Wilding et al., 2021 [13]	GLP-1 Receptor Agonists	Semaglutide	1961 (Semaglutide vs Placebo)	Nausea (44.2%), Diarrhea (31.5%), Vomiting (24.8%), Constipation (23.4%)	Nausea (17.4%), Diarrhea (15.9%), Vomiting (6.6%), Constipation (9.5%)			
Rubino et al., 2021 [6]	GLP-1 Receptor Agonists	Semaglutide	803 (Semaglutide vs Placebo)	Diarrhea (14.4%), Nausea (14%), Constipation (11.6%)	Diarrhea (7.1%), Nausea (4.9%), Constipation (6.3%)			
Rodriguez et al., 2024 [8]	GLP-1 Receptor Agonists	Semaglutide vs Tirzepatide	18,386 (Tirzepatide vs Semaglutide)	Gastroenteritis (19.75%), Cholelithiasis (11.89%), Cholecystitis (6.5%)	Gastroenteritis (20.07%), Cholelithiasis (12.66%), Cholecystitis (5.06%)			
Aronne et	GLP-1 Receptor Agonists	Tirzepatide	670 (Tirzepa	Diarrhea (10.7%), Nausea	Diarrhea (4.8%), Nausea			

al., 2024 [12]			tide vs Placebo)	(8.1%), Vomiting (5.7%)	(2.7%), Vomiting (1.2%)			
Zhao et al., 2024 [11]	GLP-1 Receptor Agonists	Tirzepatide	210 (Tirzepatide 10 mg vs 15 mg vs Placebo)	Diarrhea (40%), Nausea (30%), Vomiting (11.4%)	Diarrhea (40.8%), Nausea (32.4%), Vomiting (19.7%)	Diarrhea (8.7%), Nausea (5.8%), Vomiting (4.3%)		
Wharton et al., 2023 [7]	GLP-1 Receptor Agonists	Orforglipron	272 (Orforglipron 12 mg vs 24 mg vs 36 mg vs 45 mg vs Placebo)	Nausea (50%), Vomiting (26%), Constipation (24%), Diarrhea (24%)	Nausea (58%), Vomiting (32%), Constipation (32%), Diarrhea (36%)	Nausea (41-48%), Vomiting (14-28%), Constipation (24-28%), Diarrhea (3-14%)	Nausea (37-42%), Vomiting (27-29%), Constipation (13-19%), Diarrhea (16-33%)	Nausea (10%), Vomiting (6%), Constipation (6%), Diarrhea (10%)
Jasterboff et al., 2023 [4]	Triple-Hormone Receptor Agonists	Retatrutide	337 (Retatrutide 1 mg vs 4 mg vs 8 mg vs 12 mg	Nausea (14%), Vomiting (3%), Diarrhea (9%)	Nausea (18-36%), Vomiting (12%), Diarrhea (12%)	Nausea (17-60%), Vomiting (6-26%),	Nausea (45%), Vomiting (19%), Diarrhea (15%)	Nausea (11%), Vomiting (1%), Diarrhea (11%)

			vs Placebo)			Diarrhea (20%)	
Lundgren et al., 2021 [10]	GLP-1 Receptor Agonists	Liraglutide	195 (Liraglutide vs Exercise vs Combination vs Placebo)	Nausea (65%), Vomiting (22%), Diarrhea (27%), Constipation (18%)	Nausea (31%), Vomiting (12%), Diarrhea (15%), Constipation (15%)	Nausea (53%), Vomiting (31%), Diarrhea (29%), Constipation (24%)	Nausea (16%), Vomiting (4%), Diarrhea (8%), Constipation (12%)
Rodgers et al., 2021 [9]	GLP-1 Receptor Agonists	Exenatide	108 (Exanatide vs Diet)	Nausea (70%)	Nausea (25%)		
Cabrera-rode et al., 2023 [19]	Natural/Herbal Compound	Obex	160 (Obex vs Placebo)	Nausea in 1 case	Epigastric pain, Bloating, and Vomiting in 3 cases		
Won et al., 2024 [15]	Natural/Investigational	Vutiglabridin	12	Nausea (25%)			
Márquez-Cruz et al.,	Sympathomimetic	Phentermine	932 (Phentermine 15 mg vs 30 mg)	Abdominal pain (0.5%), Constipation	Abdominal pain (2.4%), Constipation		

2021 [3]		(3.5%), Dry mouth (6.3%), Nausea (0.5%), Vomiting (0.3%)	(4.1%), Dry mouth (13.9%), Nausea (1.1%), Vomiting (0.8%)	

The studies summarized above include both established pharmacologic agents and natural or investigational compounds. Natural products and early-phase investigational agents (e.g., Obex, Vutiglabridin, Lipigo, Gymnema sylvestre, Berberine) were included for completeness but are supported by fewer and shorter studies with smaller sample sizes and less standardized reporting compared with approved pharmaceuticals. Therefore, their evidence quality and generalizability are considered substantially lower.

References

3 Márquez-Cruz, M.; Kammar-García, A.; Huerta-Cruz, J.C.; Carrasco-Portugal, M.d.C.; Barranco-Garduño, L.M.; Rodríguez-Silverio, J.; González, H.I.R.; Reyes-García, J.G. Three- and six-month efficacy and safety of phentermine in a Mexican obese population. *Int. J. Clin. Pharmacol. Ther.* **2025**, *59*, 539–548.

4 Jastreboff, A.M.; Kaplan, L.M.; Frías, J.P.; Wu, Q.; Du, Y.; Gurbuz, S.; Coskun, T.; Haupt, A.; Milicevic, Z.; Hartman, M.L. Retatrutide Phase 2 Obesity Trial Investigators. Triple-Hormone-Receptor Agonist Retatrutide for Obesity—A Phase 2 Trial. *N. Engl. J. Med.* **2023**, *389*, 514–526.

6 Rubino, D.; Abrahamsson, N.; Davies, M.; Hesse, D.; Greenway, F.L.; Jensen, C.; Lingvay, I.; Mosenzon, O.; Rosenstock, J.; Rubio, M.A.; et al. STEP 4 Investigators. Effect of Continued Weekly Subcutaneous Semaglutide vs Placebo on Weight Loss Maintenance in Adults with Overweight or Obesity: The STEP 4 Randomized Clinical Trial. *JAMA* **2021**, *325*, 1414–1425.

7 Wharton, S.; Blevins, T.; Connery, L.; Rosenstock, J.; Raha, S.; Liu, R.; Ma, X.; Mather, K.J.; Haupt, A.; Robins, D.; et al. Daily Oral GLP-1 Receptor Agonist Orforglipron for Adults with Obesity. *N. Engl. J. Med.* **2023**, *389*, 877–888.

8 Rodriguez, P.J.; Cartwright, B.M.G.; Gratzl, S.; Brar, R.; Baker, C.; Gluckman, T.J.; Stucky, N.L. Semaglutide vs Tirzepatide for Weight Loss in Adults with Overweight or Obesity. *JAMA Intern. Med.* **2024**, *184*, 1056–1064.

9 Rodgers, M.; Migdal, A.L.; Rodríguez, T.G.; Chen, Z.-Z.; Nath, A.K.; Gerszten, R.E.; Kasid, N.; Toschi, E.; Tripaldi, J.; Heineman, B.; et al. Weight Loss Outcomes Among Early High Responders to Exenatide Treatment: A Randomized, Placebo Controlled Study in Overweight and Obese Women. *Front. Endocrinol.* **2021**, *12*, 742873.

10 Lundgren, J.R.; Janus, C.; Jensen, S.B.K.; Juhl, C.R.; Olsen, L.M.; Christensen, R.M.; Svane, M.S.; Bandholm, T.; Bojsen-Møller, K.N.; Blond, M.B.; Jensen, J.B.; et al. Healthy Weight Loss Maintenance with Exercise, Liraglutide, or Both Combined. *N. Engl. J. Med.* **2021**, *384*, 1719–1730.

11 Zhao, L.; Cheng, Z.; Lu, Y.; Liu, M.; Chen, H.; Zhang, M.; Wang, R.; Yuan, Y.; Li, X. Tirzepatide for Weight Reduction in Chinese Adults with Obesity: The SURMOUNT-CN Randomized Clinical Trial. *JAMA* **2024**, *332*, 551–560.

12 Aronne, L.J.; Sattar, N.; Horn, D.B.; Bays, H.E.; Wharton, S.; Lin, W.Y.; Ahmad, N.N.; Zhang, S.; Liao, R.; Bunck, M.C.; et al. SURMOUNT-4 Investigators. Continued Treatment with Tirzepatide for Maintenance of Weight Reduction in Adults with Obesity: The SURMOUNT-4 Randomized Clinical Trial. *JAMA* **2024**, *331*, 38–48.

13 Wilding, J.P.H.; Batterham, R.L.; Calanna, S.; Davies, M.; Van Gaal, L.F.; Lingvay, I.; McGowan, B.M.; Rosenstock, J.; Tran, M.T.D.; et al. STEP 1 Study Group. Once-Weekly Semaglutide in Adults with Overweight or Obesity. *N. Engl. J. Med.* **2021**, *384*, 989–1002.

15 Won, H.; Yoon, D.Y.; Lee, S.; Cho, J.; Oh, J.; Jang, I.; Yoo, S.; Yu, K. Effects of meal type on the bioavailability of vutiglabridin, a novel anti-obesity agent, in healthy subjects. *Clin. Transl. Sci.* **2024**, *17*, e13744.
19 Cabrera-Rode, E.; Cubas-Dueñas, I.; Acosta, J.R.; Hernández, J.C.; González, A.I.C.; Calero, T.M.G.; Domínguez, Y.A.; Rodríguez, J.H.; Rodríguez, A.D.R.; Álvarez Álvarez, A.; et al. Efficacy and safety of Obex® in overweight and obese subjects: A randomised, double-blind, placebo-controlled clinical trial. *BMC Complement. Med. Ther.* **2023**, *23*, 58.
